# The financial burden resulting from complementary and alternative medicine in oncology care

**DOI:** 10.1186/s12906-026-05417-z

**Published:** 2026-06-06

**Authors:** Sarunas Bagdonas, Louisa Schäfer, Clemens Wolf, Jutta Hübner

**Affiliations:** Klinik für Innere Medizin II, Universitätsklinikum Jena, Am Klinikum 1, Jena, 07747 Germany

**Keywords:** Complementary and alternative medicine, Oncology, Cancer, Financial burden, Financial toxicity, Out-of-pocket costs, Integrative oncology

## Abstract

**Background:**

Complementary and alternative medicine (CAM) is widely used by oncology patients to manage symptoms, reduce stress, and improve quality of life. Unlike conventional treatments, CAM is rarely reimbursed in Germany, resulting in significant out-of-pocket costs. These expenses may contribute to financial burden and financial toxicity, yet this dimension remains understudied.

**Methods:**

This multicenter, open-label, cross-sectional study was conducted in 2024. A standardized anonymous questionnaire collected demographic, socioeconomic, and medical data, CAM utilization, out-of-pocket expenditures, and distress levels (NCCN Distress Thermometer). Descriptive statistics, Chi-square tests, Pearson correlations, and independent t-tests were applied.

**Results:**

A total of 209 patients (median age 62.6 years, 52.6% female) participated; 57.4% reported CAM use. Female patients were more likely than males to use CAM (*p* < 0.001). Among CAM users, 71.7% incurred out-of-pocket expenses, averaging €124/month for women and €153/month for men. Expenditures were highest among patients with hematological malignancies (€229/month). A strong positive correlation was observed between the number of CAM modalities used and monthly costs (*p* < 0.001). No significant difference in distress was found between CAM users and non-users; however, female CAM users reported lower distress than their male counterparts (*p* < 0.05).

**Conclusions:**

CAM use is common among German oncology patients and generates substantial out-of-pocket costs, particularly in vulnerable subgroups. When combined with cancer-related income loss, these expenditures increase financial burden and may contribute to financial toxicity. Integrative oncology models, reimbursement strategies, and patient counseling are essential to mitigate economic vulnerability and ensure equitable access to supportive care.

**Supplementary Information:**

The online version contains supplementary material available at 10.1186/s12906-026-05417-z.

## Introduction

Complementary and alternative medicine (CAM) encompasses a broad spectrum of medical and health care practices that fall outside conventional treatment paradigms. Over the past decades, CAM has gained increasing acceptance among oncology patients seeking to alleviate symptoms, manage side effects, and trying to enhance their overall quality of life. Common CAM modalities include dietary supplements, herbal treatments, acupuncture, meditation, yoga, chiropractic care, and homeopathy [1]. Many patients turn to CAM as a means of supplementing standard medical therapies, reducing stress, and regaining a sense of control over their health.

The motivations for CAM use in oncology are diverse and multifaceted. Studies suggest that patients are drawn to CAM due to dissatisfaction with conventional medicine, cultural beliefs, or a preference for holistic treatment approaches. Some patients explore CAM as a last resort when conventional treatments fail, while others integrate it into their care to mitigate symptoms such as nausea, pain, and fatigue. Psychological factors also play a significant role, as CAM use is often associated with a greater sense of empowerment in treatment decision-making [2].

Most CAM services are not reimbursed by statutory health insurance, leaving patients to cover the full cost themselves. Despite widespread use, the financial consequences of CAM remain insufficiently studied. In contrast to conventional oncologic treatments, which are typically covered by health insurance or public healthcare systems, the majority of CAM therapies require direct out-of-pocket payment. This additional expense contributes to the individual financial burden and might even lead to financial toxicity, a growing concern in oncology care [3–5].

While financial burden and toxicity in oncology has been increasingly recognized, research on the economic burden of CAM remains scarce. Existing studies are limited in number and difficult to compare due to variations in healthcare systems, cultural influences, CAM accessibility, and socioeconomic factors. In Germany, in particular, research on the financial aspects of CAM use in oncology is sparse and often restricted to specific patient populations [6–9].

This study aims to address this gap by investigating the financial impact of CAM use among oncology patients. By analyzing patient demographics, expenditure patterns, and potential influencing factors, this research seeks to provide a clearer understanding of the economic burden associated with CAM. The findings may contribute to the ongoing discussion on integrating CAM into oncology care while promoting equitable access and financial sustainability.

## Methods

### Study design and ethical approval

“This multicenter, open-label, cross-sectional study was conducted using an anonymous, standardized questionnaire to assess the financial burden of complementary and alternative medicine (CAM) use among oncology patients. The study protocol and questionnaire were approved by the Ethics Committee of Jena University (Reg. Nr. 2023-3188-Bef) and received secondary approval from the Medical Association of Westphalia-Lippe. The collection of data was conducted in 2024.

### The questionnaire

The questionnaire used in this study was designed to collect the necessary data systematically. It aimed to assess key variables such as demographic characteristics, disease information, financial status and expenditures, and the use of CAM. The questionnaire was developed based on existing literature and adapted to the specific context of this study to ensure relevance and comprehensibility. Before full-scale distribution, the questionnaire was pretested on a small subset of oncology patients to assess clarity, reliability, and comprehensibility. The original questionnaire can be viewed under supplementary material.

1. Demographic and Socioeconomic Information
  Age (open-ended)
  Gender (Male / Female / Divers)
  Education level (No formal education / Primary / Secondary / Higher education)
  Family status (Alone / With partner)
  Type of health insurance (Private / Public / Other)
  Monthly household income (categorized by income brackets)
  Loss of income since the diagnosis of cancer (Yes / No, if Yes then categorized by brackets)

2. Medical History
  Diagnosis (Type of cancer and time of diagnosis)

3. Use of Complementary and Alternative Medicine (CAM)
  Current use of CAM (Yes / No)
  Types of CAM used (Multiple choice: Herbal remedies, Acupuncture, Meditation, Dietary supplements, Homeopathy, Traditional medicine, Other)
  Monthly out-of-pocket expenses for the CAM used (open-ended)
  Additional out-of-pocket expenses associated with CAM (open-ended)
  Source of information about CAM (Doctor, Internet, Friends/Family, Other)

4. NCCN Distress Thermometer
  Self-reported Distress Scale (0–10 Visual Analog Scale)
  Physical problems as source of distress (Multiple choice)

### Study population and recruitment

The study was conducted at Klinikum Lippe, a tertiary care hospital network located in Nordrhein-Westfalen, Germany. Klinikum Lippe is affiliated with the University of Bielefeld as part of the University Hospital Ostwestfalen-Lippe (UK OWL). Patients were also included from 4 affiliated private practices associated with the Department of Oncology at the University of Jena.

Inclusion criteria:

- Age ≥ 18 years
- Confirmed diagnosis of any type of cancer
- Ability to provide informed consent

Exclusion criteria:

- Presence of cognitive impairments that could interfere with survey comprehension
- Insufficient German language skills preventing completion of the questionnaire
- Incomplete survey responses leading to missing critical data

The title page of the questionnaire clearly described the objective of the study and provided participants with essential information regarding data handling. It explicitly stated that participation was voluntary and that completion of the questionnaire would be considered as informed consent for the anonymized use and analysis of the collected data for research purposes. The recruitment strategy aimed to capture a broad sample of oncology patients across different types of cancer.

### Statistical analysis

Data were anonymized and entered into a secure database for analysis. Statistical analyses were conducted using SPSS Statistics (Version 30.0) with a significance level set at *p* < 0.05.

- Descriptive statistics (means, standard deviations, frequencies) summarized demographic characteristics, CAM utilization, and financial expenditures
- Pearson’s Chi-square tests were used to analyze associations between categorical variables, such as CAM use and sociodemographic factors
- Pearson’s correlation coefficient assessed relationships between monthly CAM expenditures and the number of CAM therapies used
- Independent T-Test compared mean distress scores to evaluate statistically significant differences between CAM users and non-users and by gender

This study adheres to the ethical principles outlined in the Declaration of Helsinki and complies with all data protection regulations to ensure patient confidentiality

## Results

### Basic patient characteristics

Of the 209 patients included, the median age was 62.6 years (range 29–89). The gender distribution was relatively balanced, with 52.6% female and 47.4% male. In terms of educational background, the majority (55%) had an intermediate secondary education, 21.1% had basic secondary education, 17.7% held a university degree, and 6.2% had no formal education.

The most common cancer types were breast cancer (26.3%) and gastrointestinal cancers (22%), followed by hematological neoplasia (14.8%) and lung cancer (12%). Other cancer types included prostate cancer (7.7%), ovarian or endometrial cancer (5.3%), bladder cancer (2.9%), melanoma (1%), and other types (8.1%).

### Patient characteristics and CAM use

Among the 209 respondents, 120 patients (57.4%) reported using complementary and alternative medicine (CAM) as part of their cancer management. The analysis revealed no statistically significant differences in CAM use based on the following factors: age, marital status, educational level, type of medical insurance, monthly Net income or financial loss.

A statistically significant association was found between gender and CAM use, with female patients more likely to use CAM than male patients (Chi-Square test, *p* < 0.001) Table 1.

**Table 1:** Patient characteristics by use of CAM therapies (*n* = 209)

	CAM Use		Total *n* = 209
	No	Yes	
Median Age (years)	63	62	62,6 (29–89)
Gender, n (%)			
Female	34	76	110 (52.6%)
Male	55	44	99 (47.4%)
Total	89 (42.6)	120 (57.4)	
Educational level, n (%)			
No formal education	5	8	13 (6.2)
Basic secondary education	15	29	44 (21.1)
Intermediate secondary education	55	60	115 (55)
University degree	14	23	37 (17.7)
Family status, n (%)			
Living alone	22	34	56 (26.8)
Living with partner	67	86	153 (73.2)
Type of medical insurance, n (%)			
Private	15	17	32 (15.3)
Co-insured without contribution	1	1	2 (1)
Public insurance	72	102	174 (83.2)
Other	1	0	1 (0.5)
Monthly Net Income (Euro), n (%)			
< 1000	7	15	22 (10.5)
1001–2000	43	50	93 (44.5)
2001–2500	26	29	55 (26.3)
3001–3500	8	17	25 (12)
> 3500	5	9	14 (6.7)
Loss of Income, n (%)			
Yes	50	70	120 (57.5)
No	39	50	89 (42.5)
Los of income (Euro/month), total *n* = 120 (%)			
<100 Euro	4	8	12 (10)
100–200	9	8	17 (14.1)
201–500	16	21	37 (30.9)
501–800	10	21	31 (25.9)
801–1200	5	5	10 (8.3)
>1200	6	7	13 (10.8)

**Fig. 1:**
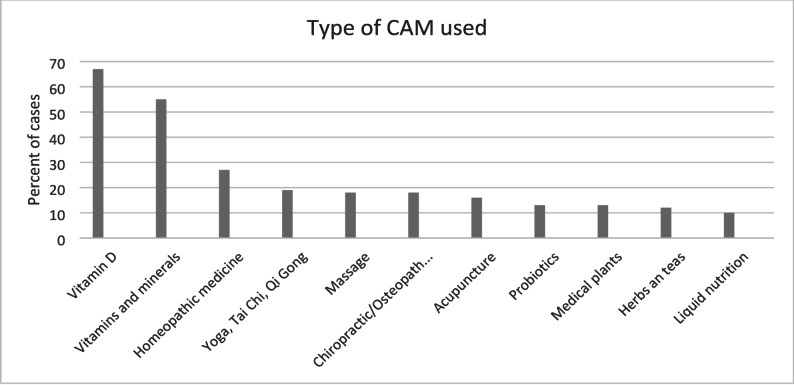
Types of CAM used (*n* = 120)

Among the 120 patients who reported using complementary and alternative medicine (CAM), the most commonly utilized therapies were vitamin D supplementation (67.7%), vitamins and minerals (55.6%), and homeopathy (27.3%). Other frequently reported CAM modalities included yoga, meditation, and relaxation techniques, as well as manual therapies such as massage, osteopathy, and chiropractic care. Additionally, some patients used acupuncture, herbal medicine, and medicinal plant extracts. CAM therapies with an incidence below 10% were omitted from the graph below Fig. 1.

**Fig. 2:**
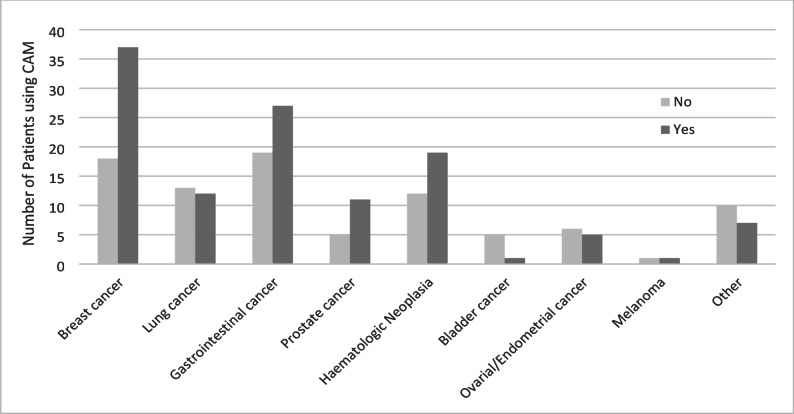
CAM use by cancer type (*n* = 209)

**Fig. 3:**
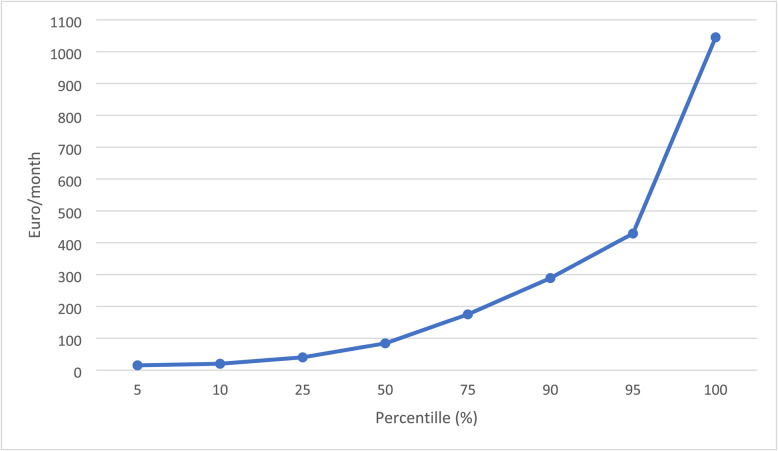
Monthly expenses for CAM (*n* = 86)

Statistical analysis confirmed that there was no significant correlation between the type of cancer and CAM use (Chi-Square test, *p* > 0.05) Fig. 2; 

To assess the financial impact of complementary and alternative medicine (CAM), only cases with direct out-of-pocket expenses were included in the analysis. Patients who reported using only free CAM modalities, such as home workouts, meditation, or self-guided relaxation techniques, or those whose CAM expenses were fully reimbursed by their insurance provider, were excluded. Among the 120 CAM users, 86 patients (71,7%) reported personal financial expenditure on CAM therapies Fig. 3. The average monthly expenditure on CAM was €153 for males and €124 for females, with no statistically significant difference between genders. The highest monthly expenditures were observed in the groups utilizing acupuncture, chiropractic/osteopathy, vitamins and minerals , and massage Table 2. 

**Table 2:** Expenditure for CAM (*n* = 120)

	No	Yes
Out of Pocket expenses for CAM	34 (28,3%)	86 (71.7%)
Mean Expenditure by CAM Type (Euro/month)		
Acupuncture	90	
Chiropractic/Osteopathy	87	
Herbs and teas	25	
Massage	74	
Probiotics	34	
Vitamins and Minerals	41	
Medical plants	20	
Homeopathic medicine	26	
Vitamin D	15	

**Fig. 4:**
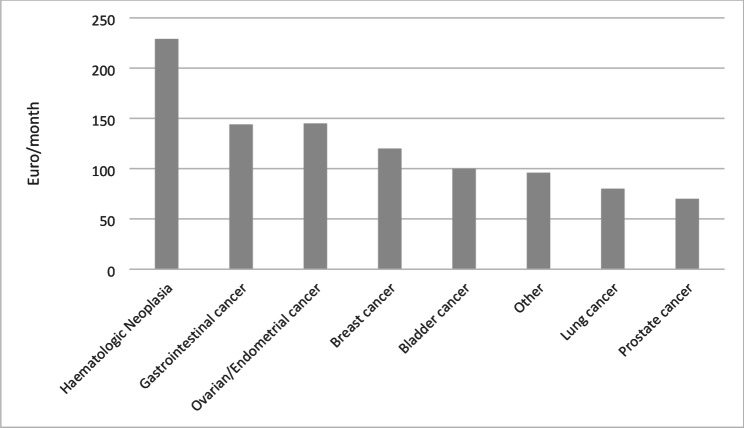
Monthly CAM expenses by type of cancer (*n* = 86)

The financial burden of CAM varied depending on the type of cancer. Patients with hematological neoplasms reported the highest average monthly CAM expenditure (€229/month), followed by gastrointestinal and ovarian/endometrial cancer (€145/month). In contrast, patients with lung cancer and prostate cancer had the lowest average CAM expenses, spending €80 and €70 per month, respectively. Among patients with hematological malignancies, the most frequently used complementary and alternative medicine (CAM) modalities were vitamins and minerals, followed by vitamin D and probiotics. In patients with gastrointestinal tumors, vitamins and minerals were also the most commonly used CAM approach, followed by herbal medicine and the use of herbs and teas. In contrast, patients with ovarian or endometrial carcinoma most frequently reported the use of massage, followed by acupuncture and vitamins and minerals Fig. 4.

**Fig. 5:**
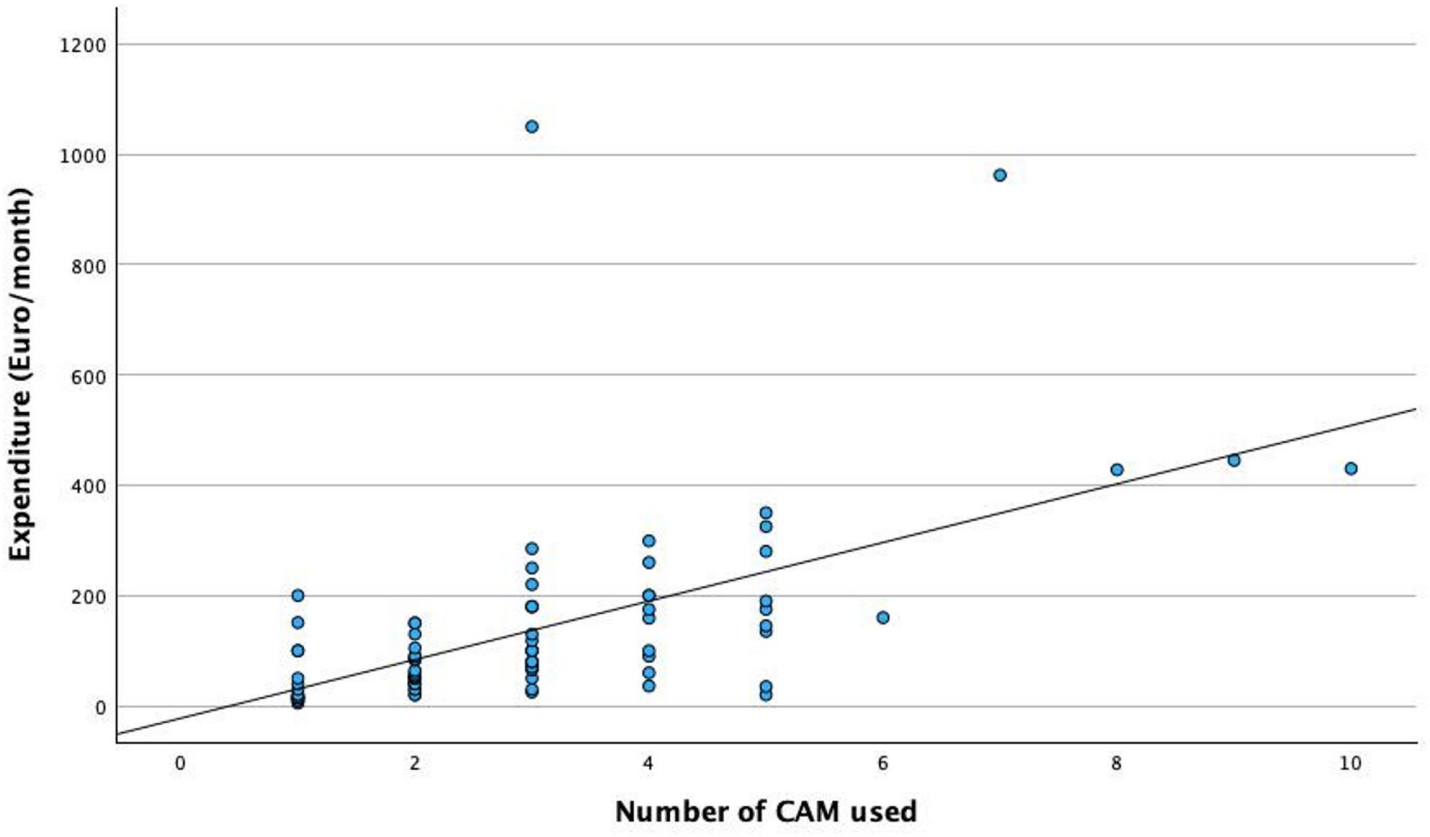
Monthly CAM expenses by number of different CAM modalities used (*n* = 86)

A statistically significant positive correlation was observed between the number of CAM modalities used and monthly CAM expenditure (bivariate Pearson correlation, *p* < 0.001) Fig. 5.

### Distress and CAM use

The mean distress scores for CAM users (5.5) and non-users (6.0) were comparable, with no statistically significant difference between the two groups (*p* = 0.394).

A statistically significant difference emerged when analyzing distress scores by gender. Among CAM users, female patients reported significantly lower distress levels compared to male patients (5.3 vs. 6.4, Independent T-Test *p* < 0.05).

## Discussion

This study provides novel and detailed insights into the phenomenon of financial burden resulting from the use of complementary and alternative medicine (CAM) among oncology patients in Germany. While CAM is frequently pursued as a form of personal empowerment, symptom management, or psychological relief, our findings demonstrate that it may carry significant, multi-layered financial risks, particularly when considered alongside cancer-related income loss. In the context of increasingly patient-centered cancer care, it is essential to critically evaluate how non-integrated and non-reimbursed supportive strategies—such as CAM—may unintentionally exacerbate economic vulnerability and psychosocial strain.

### Out-of-pocket costs and income loss as dual stressors

Among the 209 patients enrolled in this study, 57.4% reported active CAM use. While this prevalence aligns with previous international estimates [1, 10–12], our analysis highlights the substantial economic impact associated with CAM utilization. Of the CAM users, 71.7% reported out-of-pocket expenditures, averaging €124/month for women and €153/month for men. These expenses are borne almost entirely by patients themselves, as CAM is largely excluded from statutory health insurance in Germany. Only a few private insurance plans offer limited coverage for selected modalities, leaving most CAM interventions fully self-financed and thus a potential contributor to cumulative financial burden.

Importantly, these expenses must be viewed in the broader context of income loss due to illness. In our sample, 57.5% of patients experienced a reduction in income following cancer diagnosis, with nearly one-third reporting losses exceeding €500 per month. These findings are consistent with previous studies in Germany and underscore the widespread and persistent economic consequences of cancer [6–9]. Additional individual costs—such as co-payments for supportive medications or transportation to outpatient clinics—are often underreported or non-reimbursed, yet they further exacerbate patients’ financial strain [13, 14].

The combined impact of rising out-of-pocket CAM costs and declining income creates a “perfect storm” of financial burden with the possibility of financial toxicity. Patients are left with reduced economic capacity at a time when health-related expenses increase. This dual pressure undermines financial stability, compromises access to necessary services, and contributes to emotional and psychological distress [15, 16].

A prospective cohort study from Southeast Asia reported similar findings, demonstrating that a substantial proportion of net household income may be devoted to CAM use following a cancer diagnosis—and in some cases, it may even contribute to financial catastrophe [5]. Other international studies have documented wide variation in CAM-related spending, shaped by contextual factors such as healthcare system structure, cultural and religious beliefs, CAM availability, and local marketing practices [3–7, 17–19]. This variability highlights the complex, system-dependent nature of financial burden and suggests that our findings—though rooted in the German context—carry broader implications for global oncology care.

### Socioeconomic patterns and the hidden inequities of CAM use

Interestingly, our study found no statistically significant association between CAM use and traditional socioeconomic markers such as income level, educational attainment, or type of health insurance. This finding diverges from some international literature, which links CAM use more commonly to higher socioeconomic status [12, 17, 20, 21]. In the German setting, our data suggest that CAM use is not necessarily associated with financial means, but may instead reflect broader cultural, emotional, or informational drivers.

However, this apparent parity is deceptive. The similar prevalence of CAM use across socioeconomic groups may conceal deeply unequal consequences. Patients with fewer financial resources are likely to be more susceptible to long-term economic harm when incurring unreimbursed expenses for therapies with unclear clinical benefit. Without structured guidance, reimbursement options, or financial counseling, economically vulnerable patients may overextend themselves by investing in perceived holistic care at the cost of basic needs.

### Diagnosis-specific variability in CAM expenditures

A particularly notable finding of our study is the diagnosis-specific variation in CAM-related costs. Patients with hematological malignancies reported the highest average monthly expenditures on CAM (€229/month), followed by those with gastrointestinal and gynecological cancers. In contrast, prostate cancer patients reported the lowest average spending (€70/month). This 3.2-fold difference highlights the influence of disease characteristics on CAM behavior. Other studies on this topic are rare and usually focused on one diagnosis (mostly breast cancer) or general expenses; therefore, a comparison cannot be made [22].

Hematological cancers often entail chronic, cyclical treatment protocols with extensive immunosuppressive side effects, prompting patients to seek supportive strategies such as immune-enhancing supplements or fatigue-alleviating interventions. Similarly, gastrointestinal and gynecological cancers frequently involve digestive and hormonal disturbances, leading to increased interest in nutritional and hormonal regulation through CAM. Prostate cancer, often characterized by a more indolent course and managed through active surveillance, may generate a lower perceived need for such interventions.

These disparities raise important equity concerns. Patients with more treatment-intensive or burdensome diagnoses may feel a greater sense of urgency or desperation, prompting sustained investment in adjunctive therapies even when evidence is limited. In the absence of integrative oncology programs or reimbursed supportive care pathways, such patients may be especially vulnerable to financial overexposure driven by unmet needs and emotional strain.

### Multimodal CAM use and escalating financial burden

Another key finding of this study is the strong positive correlation between the number of CAM modalities used and monthly expenditure (*p* < 0.001). It is well documented that in the case of CAM use, there will often be a combination of many different modalities [23, 24]. Patients who utilized multiple CAM approaches incurred significantly higher costs. This trend suggests a dose–response relationship: the deeper a patient’s engagement with CAM, the higher their financial investment.

While some may benefit from multimodal, holistic approaches, unsupervised CAM use carries risks, including redundant therapies, potential drug interactions, and unnecessary spending [25]. Without evidence-based guidance, patients may enter a “therapeutic consumption loop”, adding successive interventions in response to unresolved symptoms or anxiety, leading to further financial and emotional exhaustion. The absence of clinical oversight in most CAM pathways makes this scenario particularly concerning.

### Gender differences in CAM use and psychological distress

Gender emerged as a significant factor in CAM behavior and psychological impact. Female patients were significantly more likely to use CAM than male patients (*p* < 0.001), consistent with literature showing greater female engagement in preventive and wellness-focused health behaviors [17, 20, 21, 26]. Among CAM users, women also reported significantly lower psychological distress than men (*p* < 0.05), as measured by the NCCN Distress Thermometer [27].

These findings suggest that CAM may offer psychological or emotional coping benefits to female patients, potentially through ritual, routine, or a sense of agency [28–30]. However, the effect was not universal; no significant differences in distress were observed between CAM users and non-users overall. This implies that CAM’s emotional impact is likely modulated by gender, individual expectations, and specific modality used, rather than representing a blanket psychological benefit.

Crucially, the interplay between distress and financial burden should not be underestimated. Emotional vulnerability may lead some patients to invest more heavily in CAM, while financial stress may in turn amplify psychological burden, especially when expectations go unmet [16, 31]. This bidirectional interaction between emotional and financial strain represents a compounded form of harmfulness that requires proactive clinical attention.

## Conclusion

CAM use among oncology patients is widespread and often perceived as supportive, empowering, or therapeutic. However, this study demonstrates that CAM is also associated with significant financial burden, particularly in the context of income loss, multi-modal use, and non-reimbursement. Our data show that financial strain is not confined to a specific demographic, but cuts across socioeconomic boundaries, with disproportionately severe consequences for those with limited resources.

The burden is not only financial. Economic hardship is closely linked to psychological distress, and may be influenced by gender, diagnosis, and the scope of CAM engagement. Without integrative care structures, clinical oversight, or financial counseling, patients are left to navigate CAM use in isolation, potentially undermining their economic, emotional, and physical well-being.

To mitigate these risks, oncology care must move toward evidence-based, financially sustainable models that integrate supportive interventions into mainstream care. Clinical teams should engage in open, nonjudgmental conversations about CAM, including discussions on cost, benefit, and affordability. Policy makers, in turn, must address reimbursement inequities and invest in regulated, accessible integrative oncology services.

In sum, recognizing and addressing the financial burden of CAM is essential to truly holistic, equitable cancer care.

### Limitations and future directions

While this study provides valuable insights, several limitations should be acknowledged:

1. Cross-Sectional Design – This study provides a snapshot of CAM use and financial burden at a single point in time. Longitudinal studies are needed to assess changes in CAM utilization, financial impact, and psychological benefits over time.
2. Self-Reported Data – The study relied on self-reported CAM use and expenditures, which may be subject to recall bias or social desirability bias. Future studies should consider validating self-reported expenses with objective financial data.
3. Lack of Specific CAM Modality Analysis – While this study categorized general CAM usage, future research should explore the individual effectiveness and cost burden of specific CAM therapies, such as vitamin D supplementation, acupuncture, or homeopathy.
4. Limited Generalizability – The study was conducted in Germany, where healthcare policies and insurance coverage for CAM differ from those in other countries. Results may not be fully generalizable to different healthcare systems.
5. Subgroups of CAM users according to cancer diagnosis varied in size, making direct comparisons difficult.
6. Data on CAM use prior to the cancer diagnosis were not collected. Therefore, it cannot be determined whether CAM utilization and related expenditures changed following the cancer diagnosis.

Future research should focus on:

- Assessing the long-term financial implications of CAM use and its role in financial burden/toxicity among oncology patients.
- Investigating the impact of specific CAM interventions on distress, quality of life, and treatment adherence.
- Exploring potential policy changes to improve insurance reimbursement for evidence-based CAM therapies.

## Supplementary Information

Supplementary Material 1.

## Data Availability

The datasets generated and analyzed during the current study are not publicly available, but are available from the corresponding author on reasonable request.

## Abbreviations

CAM: Complementary and Alternative Medicine
NCCN: National Comprehensive Cancer Network
