# Supplementary material for: The financial burden resulting from complementary and alternative medicine in oncology care
Source: BMC Complement Med Ther. 2026 Jun 6;26:205. doi: 10.1186/s12906-026-05417-z (PMC13242131; doi:10.1186/s12906-026-05417-z)
Supplement: Supplementary file 1 — Supplementary Material 1. [file 12906_2026_5417_MOESM1_ESM.docx]

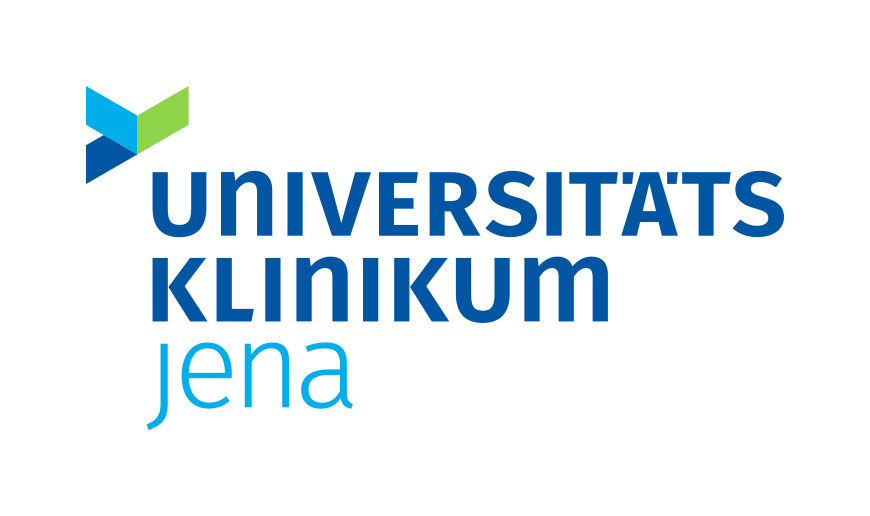


**The Financial Burden Resulting from**

**Complementary and Alternative Medicine in Oncology Care**

Dear Participants,

Cancer is a serious disease that affects not only the physical and psychological well-being of those affected, but may also have significant financial consequences. In particular, the costs associated with complementary therapies and alternative medicine can represent a substantial burden. With this survey, we aim to gain insight into your personal experiences and perceptions regarding this issue.

In this survey, anonymized data will be collected, stored, and analyzed. The use of the data will be carried out in accordance with legal regulations and requires your consent:

1. I agree that anonymized data about me will be collected as part of this survey and stored in paper form as well as on electronic data carriers of password-protected computers for scientific analysis.
2. I have been informed that my participation in the survey is voluntary and that I provide my consent. Consent to the collection and processing of my anonymized data is irrevocable. Due to the anonymized nature of data collection, the stored data cannot be deleted retrospectively.

By completing and submitting or returning the questionnaire, you consent to participation in the survey.

Completing the questionnaire will take approximately 10–15 minutes of your time.

**Study Coordination:**

Prof. Dr. med. Jutta Hübner
Department of Internal Medicine II, Hematology and Medical Oncology, Jena University Hospital
E-mail: jutta.huebner@med.uni-jena.de

Sarunas Bagdonas
Department of Internal Medicine II, Hematology and Medical Oncology, Klinikum Lippe
E-mail: sarunas.bagdonas@klinikum-lippe.de

1. **What gender are you?**  Male  Female  Diverse

# How old are you? ______ years

1. **What type of tumor disease do you have?** ___________________
2. **Since when has your tumor diagnosis been known?** ______/________ (month/year)

# How many people currently live in your household?

 I live alone  I live together with my Partner/Spouse

Minor children living in the household: ___________ (number)

# Highest level of education completed

 No formal qualification  Secondary school certificate

 University entrance qualification  University degree

# Would you tell us your monthly net income?

 under 1.000 Euro  2.501 – 3.000 Euro

 1.001 – 2.000 Euro  3.001 – 3.500 Euro

 2.001 – 2.500 Euro  over 3.500 Euro

# What type of health insurance do you have?

 Private   Statutory (public)

 Insured free of charge as a dependent  Other, please specify: ___________

# Have you suffered any loss of income since the diagnosis of your tumor disease?

 Yes  No

# If yes, how much per month?

 less than 100 Euro  501 - 800 Euro

 100 - 200 Euro  801 - 1,200 Euro

 201 - 500 Euro  over 1,200 Euro

# If yes, did you have the opportunity to compensate for these losses?

(Multiple answers possible)

 No  Yes, via credit

 Yes, via savings or assets  Yes, other: ____________

1. **Has your spouse changed his/her employment relationship since your cancer diagnosis to compensate for your loss of income?**

 works more  works less  unchanged

**Complementary and Alternative Medicine (CAM)**encompasses a range of treatment methods and therapies that may be used in addition to or as an alternative to conventional medical approaches. CAM includes a wide variety of therapies, such as vitamins and minerals, herbal medicine, acupuncture, massage, homeopathy, meditation, Ayurveda, cancer-specific diets, and many others.

1. **Are you currently using complementary and alternative therapy?**

 Yes  No

If yes, how much per month do you spend on the following methods/procedures?

| **Methods / Interventions** | **Used** | **Monthly expenses:** |
| --- | --- | --- |
| Vitamin D |  |  |
| Other vitamins, minerals and/or secondary plant compounds |  |  |
| Mistletoe therapy |  |  |
| Medicinal plants: __________________ |  |  |
| Enzyme preparations to reduce side effects of anticancer medications |  |  |
| Chinese or Ayurvedic herbs/teas and medicinal plants |  |  |
| Homeopathy, Schuessler salts or Bach flowers |  |  |
| Probiotics |  |  |
| Detoxifying procedures |  |  |
| Laying on of hands (e.g. Reiki and similar) |  |  |
| Acupuncture/ Acupressure |  |  |
| Massage or reflex therapy |  |  |
| Chiropractic therapy/Osteopathy |  |  |
| High-calorie oral nutritional supplements |  |  |
| Special cancer diet (e.g. fasting, ketogenic or low-carb diet) |  |  |
| Changing the diet to vegetarian or vegan: |  |  |
| Sports / exercise at home or outdoors (e.g. running, Nordic walking) |  |  |
| Membership in a sports club |  |  |
| Rehabilitation sports |  |  |
| Yoga / Tai Chi / Qi Gong |  |  |
| Other: ___________________ |  |  |

1. **Are there any additional costs associated with your CAM treatment? (You may select more than one option)**

 Travel costs to the CAM treatments: _____________/month

 Fees/charges for non-medical practitioners or other physicians: ___________/month

 I am driven/accompanied to the CAM treatments: ______ times/month

 Relatives/friends take a day off to accompany me ______ times/month

1. **Who recommended the CAM therapy/application to you? (You may select more than one answer)?**

 Family doctor  Oncologist  Pharmacist  Friends/family  Internet research

# How would you rate the burden caused by your illness?

1. Please circle the number (0-10) that best describes how much distress you have been experiencing in the past week including today


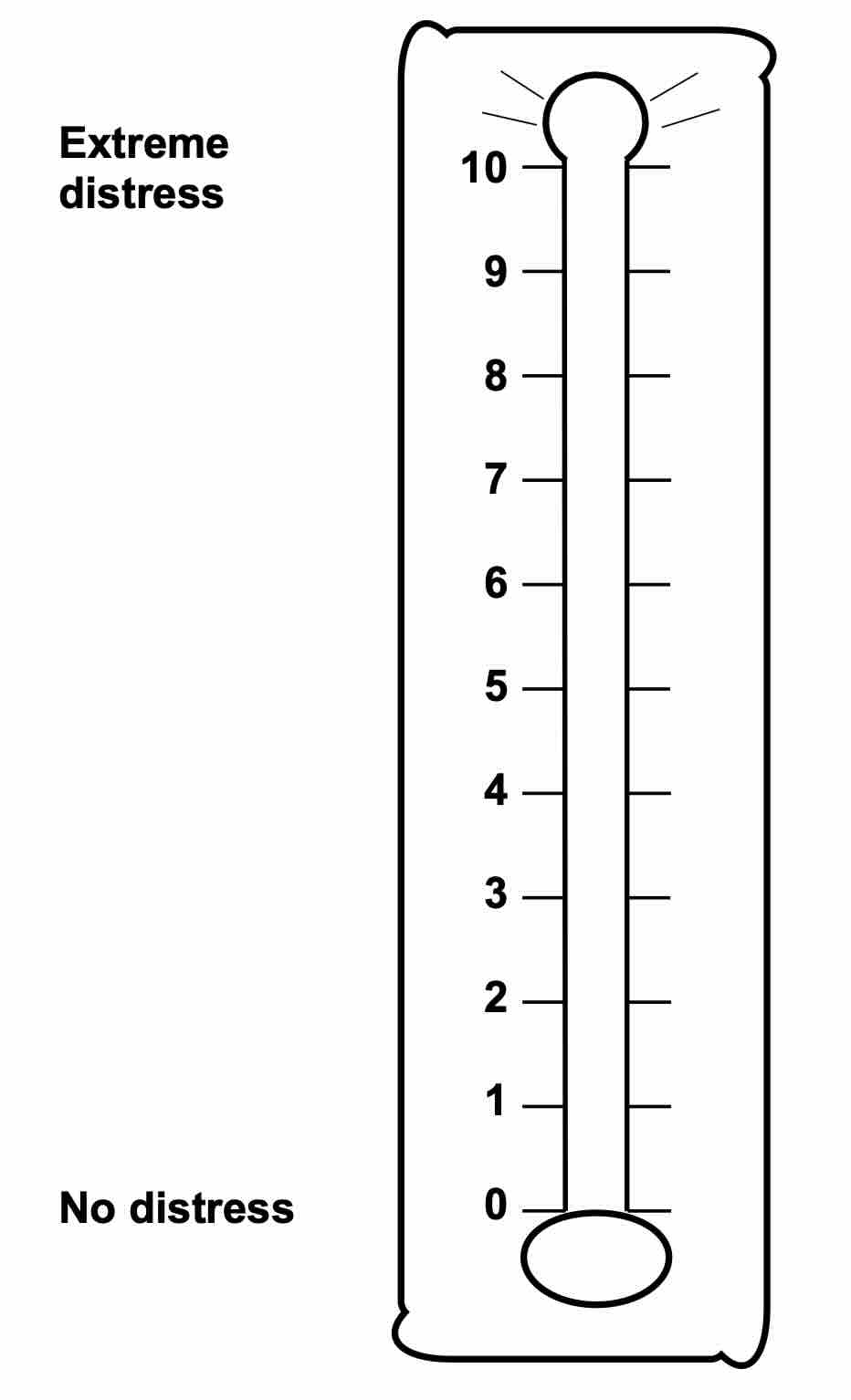


Thank you for participating!

1. Please indicate if you have had any issues in any of the following areas in the last week, including today.

| **Physical problems** | | |
| --- | --- | --- |
| Yes | No |  |
|  |  | Food/Nutrition |
|  |  | Nausea |
|  |  | Inflammation in the mouth area |
|  |  | Indigestion |
|  |  | Constipation |
|  |  | Diarrhea |
|  |  | Fever |
|  |  | Exhaustion |
|  |  | Breathing |
|  |  | Pain |
|  |  | Urination |
|  |  | Sleep disorder |
|  |  | Washing, dressing |
|  |  | Movement/Mobility |
|  |  | External appearance |
|  |  | Dry/itchy skin |
|  |  | Dry/stuffy nose |
|  |  | Tingling in hands/feet |
|  |  | Feeling bloated |
|  |  | Sexual Problems |
